# Simultaneous Determination and Pharmacokinetics of Peimine and Peiminine in Beagle Dog Plasma by UPLC-MS/MS after the Oral Administration of *Fritillariae ussuriensis* Maxim and *Fritillariae thunbergii* Miq Powder

**DOI:** 10.3390/molecules23071573

**Published:** 2018-06-28

**Authors:** Zhibin Wang, Feng Cao, Yajun Chen, Zhenqiu Tang, Zhenyue Wang

**Affiliations:** School of Pharmacy, Heilongjiang University of Chinese Medicine, 24 Heping Road, Xiangfang District, Harbin 150040, Heilongjiang, China; wzbmailbox@hljucm.net (Z.W.); 18435166854@163.com (F.C.); 18846925019@163.com (Y.C.); 13199472861@163.com (Z.T.)

**Keywords:** *Fritillaria*, dog plasma, isosteroidal alkaloids, UPLC-MS/MS, pharmacokinetics

## Abstract

A simple and high sensitive ultra-high performance liquid chromatography-tandem mass spectrometry (UPLC-MS/MS) method was developed and validated for the simultaneous determination of peimine and peiminine in beagle dog plasma after the oral administration of *Fritillariae ussuriensis* Maxim and *Fritillariae thunbergii* Miq powder. Chromatographic separation was achieved on an ACQUIT UPLC^®^ BEH C_18_ column (1.7 μm, 2.1 × 100 mm) in a gradient elution way with a mobile phase consisting of acetonitrile and water containing 0.1% formic acid at a flow rate of 0.4 mL/min. The plasma samples were prepared by a liquid–liquid extraction (LLE) method with ethyl acetate. The analytes were detected with a triple quadrupole tandem mass spectrometry (MS) in multiple reaction monitoring (MRM) mode and a positive ion electrospray ionization (ESI) of the transitions at *m*/*z* 432.4→414.4 for peimine and *m*/*z* 430.3→412.3 for peiminine. The method was linear for two analytes over the investigated range with all determined correlation coefficients exceeding 0.9900. The lower limit of quantification (LLOQ) was 0.988 ng/mL for peimine and 0.980 ng/mL for peiminine. The mean extraction recoveries of peimine and peiminine at three quality control samples (QC) levels were ranged from 82.56 to 88.71%, and matrix effects ranged from 92.06 to 101.2%. The intra-day and inter-day precision and accuracy were within the acceptable limits at LLOQ and QC levels. The method was effectively and successfully applied to the pharmacokinetics of peimine and peiminine after oral administration of powder to beagle dogs. The obtained results may be help to guide the clinical application of *Fritillaria ussuriensis* Maxim and *Fritillaria thunbergii* Miq.

## 1. Introduction

*Bulbus fritillaria* is recorded with the Chinese name “Bei Mu” in classical Chinese medicine books, and has been used as a traditional medicine for thousands of years [1]. *Bulbus fritillaria* is the dried bulbus of *Fritillaria ussuriensis* Maxim (**FU**) or *Fritillaria thunbergii* Miq (**FT**) in the genus *Fritillaria* (the Liliaceae family) [2,3]. **FU**, known as “Ping-beimu,” is recorded officially, and it has been previously reported that a number of alkaloids are major biologically active components [4]. **FT** is often referred to as “Zhe-beimu” [5]. This plant is mainly grown in the Heilongjiang, Zhejiang ,and Jiangsu provinces of China. Peimine and peiminine are selected as the phytochemical marker compounds of **FU** and **FT** for quality control according to the 2015 edition of *The Pharmacopoeia of the People’s Republic of China*. Therefore, in this study, peimine and peiminine were selected as bioactive compounds for the determination.

**Figure 1:**
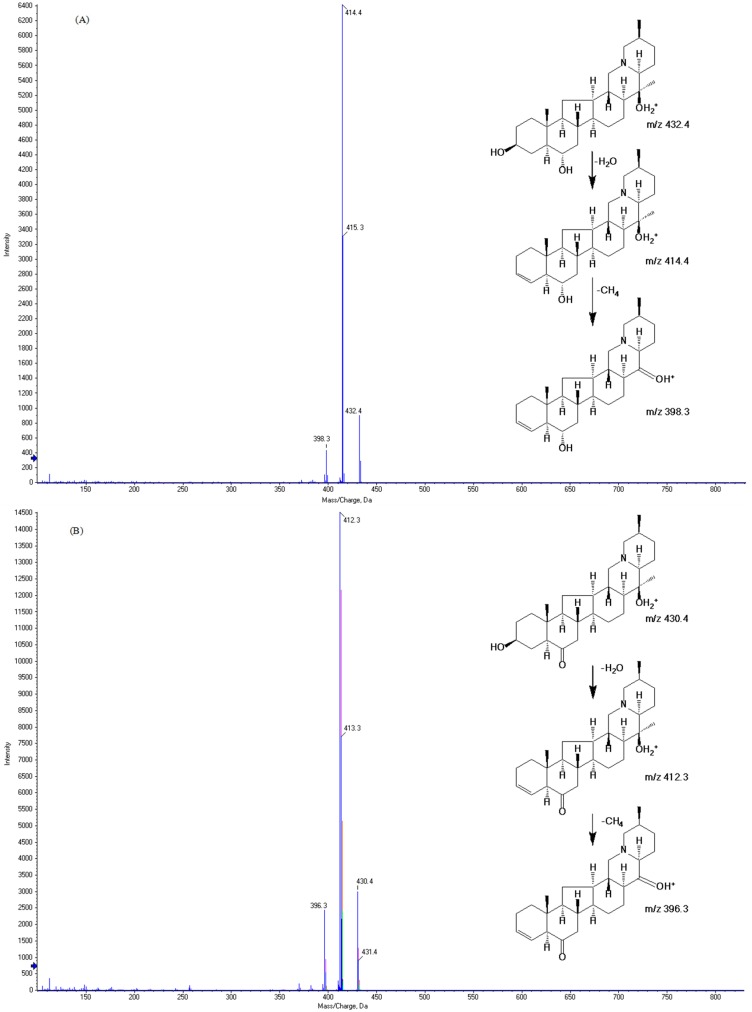
MS/MS fragmentation patterns of peimine (**A**) and peiminine (**B**).

Peimine and peiminine are isosteroidal alkaloids and the major biological active constituents of **FU** and **FT** (Figure 1) [6]. Various pharmacological effects of peimine and peiminine have been reported [7], such as anti-inflammatory [6,8], anti-tumor [9,10,11], antioxidant [12], antitussive, and sedative [13] effects. Peimine inhibits the production of pro-inflammatory cytokines, such as IL-6, IL-8, and TNF-α. It also reduces the phosphorylation MAPKs and NF-κB expression in PMACI-induced HMC-1 cells [14]. In an anti-inflammatory evaluation assay, peimine was able to block the Nav1.7 ion channel and preferably inhibited the Kv1.3 ion channel [6]. Peiminine has been reported to be used in the treatment of atopic dermatitis (AD) and Parkinson’s disease (PD). Peiminine protects dopaminergic neurons in the (Lipopolysaccharide) LPS-induced PD model by inhibiting neuroinflammation [15]. A previous study showed that peiminine had inhibitory effects on inflammatory cytokines in HMC-1 cells and on anaphylactic shock [16]. Peiminine attenuates the expression of inflammatory cytokine in cells including RAW 264.7, HaCaT, and RBL-2H3 cells, and can be used for the treatment of AD [17].

Several analytical methods have been used in the qualitative or quantitative determination of peimine and peiminine [18], including ultra-high performance liquid chromatography tandem mass spectrometry (UHPLC-MS/MS), rapid liquid chromatography tandem mass spectrometry (UPLC-TQ/MS), gas chromatography coupled with flame ionization detector (GC-FID), electronic ionization mass spectrometry (EI-MS), high-performance liquid chromatography coupled with ultra-violet visible detector (HPLC-UV), capillary electrophoresis (CE), and HPLC coupled with evaporative light scattering detection (HPLC-ELSD) [19,20,21,22,23]. Among these methods, HPLC-UV is not suitable for a large number of sample analyses; the CE and HPLC-ELSD methods show a lack of sensitivity. While these methods mainly focused on the quantification of peimine in raw materials or pharmaceutical preparations, to our knowledge, no method has been reported on the simultaneous determination and pharmacokinetics of both peimine and peiminine in *Fritillaria ussuriensis* Maxim (**FU**) in a single run. Therefore, it is of great interest to develop a specific, simple method to simultaneously determine the main components of **FU** and **FT** in beagle dog plasma, which could be applied to compare the pharmacokinetic characteristics of **FU** and **FT** in subsequent studies.

In this study, we developed a rapid, high-sensitivity, high-performance liquid chromatography–mass spectrometry (UPLC-MS/MS) combined multiple reaction monitoring (MRM) technique for the simultaneous determination of peimine and peiminine in beagle dog plasma for the first time. The validated method has been successfully developed for the pharmacokinetic study of peimine and peiminine in **FU** and **FT** powder.

## 2. Results and Discussion

### 2.1. Method Development

#### 2.1.1. UPLC-MS/MS Optimization

The experiment was performed on an ACQUIT UPLC^®^ BEH column (1.7 μm, 2.1 × 100 mm, Waters, Milford, MA, USA) link to an AB Sciex QTRAP^®^ 3200 (AB Sciex, Toronto, ON, Canada) equipped with an ESI interface for maximum specificity and sensitivity. We employed negative ionization mode to obtain the precursor and product ions of the analytes; the response of the two analytes were very bad. However, all analytes were more efficiently ionized in the positive-ion mode than in the negative-ion mode [24]. For MS/MS detection, we used electrospray ionization (ESI) source in the positive-ion mode. The optimized parameters are as follows: curtain gas, 10 psi; ion source gas 1, 55 psi; Ion source gas 2, 55 psi; source temperature, 450 °C. The optimized mass transitions ion pairs were monitored using MRM mode of transitions from precursor ions to product ions at *m*/*z* 432.4→414.4 for peimine, *m*/*z* 430.3→412.3 for peiminine, *m*/*z* 181.2→124.1 for theophylline. The product ions of peimine at *m*/*z* 414.4 and peiminine at *m*/*z* 412.3 were detected after both losing H_2_O. All the product ions were chosen according to the stability and ion response in previous studies [18,25,26,27,28,29]. The innovation of this study was that qualifier ions of peimine at *m*/*z* 398.3 and peiminine at *m*/*z* 396.3 were detected after losing both H_2_O and –CH_4_. The two analytes yielded protonated molecular ions ([M + H]^+^) as the base peak. The parameters of the fragmentor and collision energies were optimized in order to obtain the richest relative abundance of precursor and product ions. The optimization of the parameters was sensitive and stable, and could be applied to pharmacokinetic investigation of peimine and peiminine [30]. Theophylline was used as the internal standard (IS) as it exhibited a stable response, effective separation, and made this analytical method more accurate.

The optimized mass spectrometric parameters for the two analytes and IS are shown in Table 1. The study used the standard of the analytes and IS to obtain the maximum sensitivity of the MRM mode. The chemical structure and MS fragmentation patterns of peimine and peiminine are shown in Figure 1.

In order to produce sharp peak shape, enhance ESI, and obtain adequate responses for two analytes and IS, initial feasibility experiments on various mixtures of mobile phase such as acetonitrile–water and methanol–water were performed, and different buffers including acetic acid, formic acid, and ammonium acetate were evaluated. The results showed that a mobile phase of acetonitrile–water exhibited satisfactory peak shape and sensitivity. In addition, 0.1% formic acid was used to improve the peak symmetry and response. Hence, acetonitrile and 0.1% formic acid in water was selected as the best solvent mixture. The final separation was accomplished in a gradient elution way using an ACQUIT UPLC^®^ BEH C_18_ column (1.7 μm, 2.1 × 100 mm) at 40 °C including acetonitrile-water (0.1% formic acid) at a flow rate of 0.4 mL/min. The injection volume was 2 μL. The selected conditions were found to be suitable for all analytes that can be rapidly separated within 3 min, without interference from endogenous components in beagle dog plasma.

#### 2.1.2. Selection of the Internal Standard and the Extraction Method

To obtain high extraction recovery, both protein precipitation by methanol and acetonitrile in plasma, and liquid–liquid extraction (LLE) methods using several kinds of solvents including dichloromethane, ethyl acetate, and methyl tert-butyl ether have been evaluated during sample preparation in the study. In the dichloromethane treatment process, the lower layer was tied up, and impurities were easily brought in samples. The other extraction recovery was low, so ethyl acetate was selected as the extraction solvent. The analytes could be extracted effectively in plasma and the response of the two analytes was higher with liquid–liquid extraction by ethyl acetate. Additionally, ethyl acetate can easily volatilize under nitrogen gas at 40 °C and is environmentally friendly. Finally, plasma samples were pretreated with methanol precipitation combined with ethyl acetate extraction. The extraction recoveries were high—82.5%. In order to find a suitable IS monitoring the analytes, we screened several alkaloids, including phenacetin, berberine, dapsone, and theophylline. All compounds can be separated with good resolution, but these had their disadvantages. Phenacetin, berberine, and dapsone had a longer retention time and interference in plasma matrix. Theophylline was selected as the IS because of the suitable retention time and great precision. In addition, theophylline had a satisfactory peak shape in the chromatographic separation and had no obvious interference in matrix.

### 2.2. Method Validation

#### 2.2.1. Selectivity

**Figure 2:**
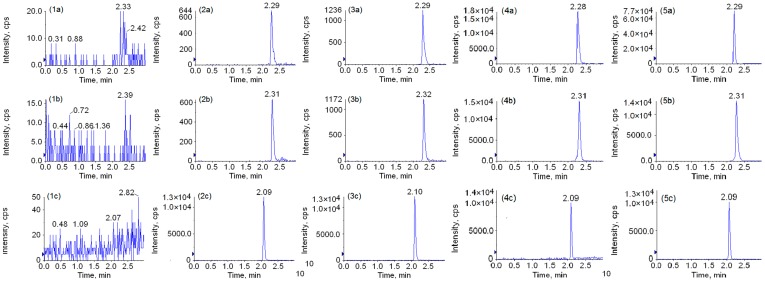
Representative MRM chromatograms of peimine (**a**), peiminine (**b**) and IS (**c**) in beagle dog plasma samples. Blank beagle dog plasma (**1**), a spiked plasma sample with the analytes at LLOQ (**2**), a blank plasma with IS and analytes at low QC levels (1.976 ng/mL for peimine, 1.960 ng/mL for peiminine) (**3**), a plasma sample taken 2 h after oral administration of 1.0 g/kg *Fritillaria ussuriensis* Maxim powder (**4**) and 1.0 g/kg *Fritillaria* *thunbergii* Miq powder (**5**).

The retention time of peimine, peiminine, and IS was about 2.29, 2.31, and 2.09 min. Theophylline is a methyl purine alkaloid, while peimine and peiminine are isosteroidal alkaloids. Compared with the analytes, the molecular mass of theophylline is smaller and the polarity is larger. In reverse phase chromatography, the IS eluted prior to the analytes because of the polar first-in peak. The representative MRM chromatograms were obtained from blank beagle dog plasma (A), a spiked plasma sample with the analytes at LLOQ (B), blank plasma with the I.S. and analytes at low QC levels (1.976 ng/mL for peimine, 1.960 ng/mL for peiminine) (C), a plasma sample taken 2 h after oral administration of 1.0 g/kg **FU** powder (D) and 1.0 g/kg **FT** powder (E), respectively. As shown in Figure 2, the IS eluted prior to the analytes; the overlapping peaks of the signal and interference signal from endogenous substances were not detected at the retention time of the analytes and IS from the beagle dog plasma samples. Therefore, this method was successfully applied for the determination of two compounds in the dog plasma matrix.

#### 2.2.2. Linearity and Lower Limit of Quantification

The linearity of the method was determined by analyzing a series of standard samples with different concentrations. The regression equation, correlation coefficients and linear ranges for the two analytes are shown in Table 2. The abscissa *X* represents the plasma concentration of analytes. The ordinate *Y* represents the peak area ratio of analytes to I.S. The linear calibration ranges were 0.988–197.6 ng/mL for peimine and 0.980–196.0 ng/mL for peiminine. The LLOQ for peimine and peiminine were 0.988 and 0.980 ng/mL, respectively. The standard calibration curves of two analytes were linear, as the values of the correlation coefficients exceeded 0.9900 (0.9988 for peimine; 0.9990 for peiminine). The range of calibration was sufficient for quantitative determination of two analytes after oral administration of **FU** and **FT** powder to beagle dogs.

#### 2.2.3. Precision and Accuracy

The intra-day and inter-day precision and accuracy of the method were assessed at LLOQ and QC levels (0.988/1.976/39.52/197.6 ng/mL for peimine and 0.980/1.960/39.20/196.0 ng/mL for peiminine, respectively) in six replicates on the same day and on three consecutive validation days. The results are listed in Table 3. The intra-day and inter-day precision (RSD, %) of the two analytes were in the ranges of 7.40–14.47% and 3.77–14.40%, respectively. The accuracy ranged from −5.57 to 7.37%. Both RSD and RE were typically within the requirements (<15%) defined by regulatory guidelines for all analytes. Thus, the established method was precise and accurate.

#### 2.2.4. Extraction Recovery and Matrix Effects

Extraction recovery and matrix effect of the two alkaloids were evaluated in the plasma. The results are depicted in Table 4. The extraction recoveries of peimine and peiminine at three QC levels (LQC, MQC, HQC) were in the range of 82.56–85.09% and 83.08–88.71%, respectively. The matrix effects were 93.34–98.74% for peimine, 92.06–101.2 for peiminine, and 93.36% for IS. The mean extraction recovery of IS was 96.57% at 1530 ng/mL. Indicating that the recoveries of three analytes were consistent and there was no endogenous substances interfering with the ionization of analytes and IS.

#### 2.2.5. Stability

Triplicate beagle dogs plasma samples at low, medium and high concentrations were determined under a variety of storage conditions. The stability studies were investigated in four storage conditions. The results in Table 5 indicate that the two analytes are stable in spiked plasma under various experimental conditions. All the analytes were stable at −20 °C for at least one month. The RSD values in all stability studies were ranged from 12.20 to −6.34%, which met the criterion for stability measurements.

### 2.3. Pharmacokinetic Study

The UPLC-MS/MS method developed was successfully applied to simultaneously determine the two analytes in plasma after a single oral administration of 1.0 g/kg of powder to six beagle dogs [31]. The mean plasma concentrations of the two analytes were determined for a series of time points, and the data obtained from six dogs were averaged. We chose the oral dosage for the dogs corresponding to the human dosage in the *Chinese Pharmacopoeia* (2015 edition).

There are several reports on assaying peimine and peiminine in *Bulbus Fritillaria* [18,19,23,31], but no analytical method was developed for the simultaneous determination of the given two alkaloids in **FU** and **FT** powder, Most studies mainly focused on the determination of several other alkaloids present in high contents. Liu et al. developed a method to determine the two alkaloids simultaneously from alkaloid extracts of *Bulbus Fritillaria* in rat plasma [30]. In this method, the LLOQs were 1.0 ng/mL for both peimine and peiminine and the run time was 11.2 min. There was another report on the simultaneous determination of two alkaloids from alkaloids extracts of **FT** and **FT**—*Glycyrrhiza uralensis* Fisch [18] couple extract in rat plasma. The LLOQ for both of peimine and peiminine was 1.0 ng/mL and the run time was 5.4 min. The chromatographic run time in this study was 3 min. In comparison, we used oral administration to beagle dogs; the method described in this study is faster than other methods and is more suitable for clinical applications. **FT** and **FU** is the main herb in TCM prescriptions such as Haizao Yuhu decoration, Taiping Shenghui Fang, Puji Fang, and Shengji Zonglu. To improve the understanding of the difference between pharmacokinetics and even pharmacology of **FU** and **FT**, a simple and highly sensitive analytical method for the simultaneous determination of the main bioactive components in two herbs after oral administration of TCM in plasma is required. To our best knowledge, this is the first time peimine and peiminine have been determined in **FU** simultaneously using an UPLC-MS/MS method. Also, this is the first time the pharmacokinetic characteristics of **FU** and **FT** have been compared after oral administration to beagle dogs in subsequent studies. 

**Figure 3:**
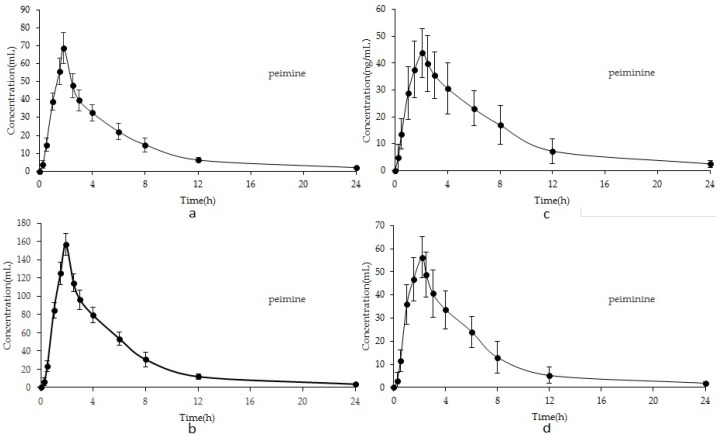
Mean concentration–time profiles of peimine of **FU** (**a**) and **FT** (**b**); peiminine of **FU** (**c**) and **FT** (**d**).

The mean plasma concentration–time curves are illustrated in Figure 3. The corresponding pharmacokinetics parameters, including the half-time (*T*_1/2_), elimination rate constant (*Ke*), area under concentration–time curve (AUC_0-t_, AUC_0-∞_), maximum plasma concentration (*C*_max_), and time to reach the maximum concentration (*T*_max_), are listed in Table 6.

The *T*_max_ values of peimine and peiminine of **FU** were 1.83 and 2.08 h. the *T*_max_ values of peimine and peiminine of **FT** were achieved in 1.91 and 2.17 h, respectively. All analytes achieved their *C*_max_ in plasma within 1.5–2.2 h after oral administration, which was consistent with a previous study about oral administration and suggested that the absorption of peimine in vivo was fast (less than 2 h) [30]. That the *T*_max_ values of peimine and peiminine were different might be due to the different substituent groups’ structure, which is hydroxyl for peimine and carbonyl for peiminine. The *T*_1/2_ values were 3.012 ± 0.16, 4.453 ± 0.35 for peimine and peiminine, respectively, in **FU**. The *T*_1/2_ values were 2.817 ± 0.11, 2.965 ± 0.06 for peimine and peiminine, respectively, in **FT**. The *T*_1/2_ valued varied in two analytes. It suggested that the elimination of the two analytes was different in vivo. The difference between peimine and peiminine might be because of the different substituent groups’ structures [24]. Moreover, the AUC_0-t_ after oral administration ranged from 340.1 ± 16.86 to 759.4 ± 11.12 for peimine and from 319.8 ± 12.54 to 323.7 ± 39.19 for peiminine. The AUC_0-∞_ ranged from 367.2 ± 22.35 to 808.2 ± 14.91 for peimine and from 342.1 ± 16.11 to 370.1 ± 49.34 for peiminine. The rank order of AUC_0-t_ and AUC_0-∞_ was peimine > peiminine, which corresponded to administration dosages. The parameters indicate that the absorption, distribution, and elimination of peimine are faster than those of peiminine. These results should be helpful for further studies on the pharmacokinetics, pharmacy, and toxicity of **FU** and **FT** and should promote research into the efficacy of the two herbs in clinical therapeutic studies.

## 3. Materials and Methods

### 3.1. Materials and Reagents

Reference standards of peimine (Must-17021101) and peiminine (Must-17050511) were purchased from Chengdu Must Bio-Technology Co., Ltd. (Chengdu, China). The internal standard (IS), theophylline was purchased from the National Institutes for Food and Drug Control (100121-199903, Beijing, China). Both acetonitrile and methanol of HPLC grade were purchased from Concord Technology Co., Ltd. (Tianjin, China). Acetic acid of HPLC grade was obtained from Thermo Fisher Scientific (Shanghai, China). Ethyl acetate of analytical grade was purchased from Guangzhou Fan Hong Trade Co., Ltd. (Guangzhou, China). Ultrapure water was prepared using a Millipore Milli-Q purification system (Millipore, Molsheim, France). Drug-free dog plasma and QC samples were prepared from six different dogs and stored at −20 °C until use.

Two herbs, the dried bulbus of *Fritillaria ussuriensis* Maxim and *Fritillaria thunbergii* Miq, were purchased from Anguo (Hebei, China), one of the largest Chinese herbal medicine distribution centers in the world, in September, 2016, and identified by Professor Lianjie Su of Heilongjiang University of Chinese Medicine.

### 3.2. Preparation of FU Powder and FT Powder

**FU** and **FT** were crushed to pieces before use. The powder passed through a No. 5 sieve and the powder could pass No. 6 sieve no less than 95%. *Fritillaria ussuriensis* Maxim dried powder 92 g was obtained and *Fritillaria thunbergii* Miq was obtained 96 g. The two analytes’ contents in both powders were measured quantitatively by HPLC-ELSD. The content of peimine was 0.58 mg/g and peiminine was 0.72 mg/g in **FU** powder. Meanwhile, the content of peimine was 1.2 mg/g and peiminine was 0.87 mg/g in **FT** powder.

### 3.3. Instruments and Chromatographic Conditions

The UPLC-MS system (Waters Technologies series, MA, USA) coupled to an AB Sciex 3200 triple quadrupole tandem mass spectrometer (AB Sciex, Toronto, ON, Canada) equipped with an electrospray ionization (ESI) interface (Framingham, MA, USA). Chromatographic separation was performed using a C_18_ column (ACQUITY UPLC^®^ BEH C_18_ column, 1.7 μm, 2.1 × 100 mm) in a gradient elution way, composed of (A) acetonitrile and (B) water containing 0.1% formic acid at a flow rate of 0.4 mL/min. The gradient elution started at 5% A and 95% B, followed by a linear increase of solution A to 70% within 0.5 min. The condition of 70% A was maintained for 2.5 min, finally the gradient decrease to 5% A within 0.5 min. The analysis time was 3 min for each injection. The column temperature was set to 40 °C. A 2 μL aliquot of the sample solution was injected into the system.

The quantification was obtained in the multiple reactions monitoring (MRM) mode with the positive ionization mode at a source temperature of 450 °C. The nebulizing gas was high-purity N_2_, which was used as the drying gas at a flow rate of 11.0 L/min at 40 °C. The precursor-product ion transition was at *m*/*z* 432.4→414.4 for peimine, *m*/*z* 430.3→412.3 for peiminine, and *m*/*z* 181.2→124.1 for theophylline, respectively. The fragment was at 124 V for peimine, 101 V for peiminine, and 62 V for theophylline, respectively.

### 3.4. Preparation of Calibration Standards and Quality Control Samples

The mixed stock solutions of peimine and peiminine were obtained at concentrations of 49.4 μg/mL and 49.0 μg/mL, respectively, and further diluted in methanol to obtain peimine and peiminine working solutions with a series of concentrations. The standard solution samples for the standard calibration curves were prepared at final concentrations of 0.988, 1.976, 9.880, 19.76, 39.52, 98.80, and 296.4 ng/mL for peimine; and 0.980, 1.960, 9.800, 19.60, 39.20, 98.00, and 294.0 ng/mL for peiminine. The IS working solution was prepared at a concentration of 1530 ng/mL by diluting the stock solution with methanol. Four concentration levels of QC samples were prepared in the appropriate matrix blank plasma at nominal concentrations of high QC (197.6/196.0 ng/mL), medium QC (39.52/39.20 ng/mL), low QC (1.976/1.960 ng/mL), and LLQC (0.988/0.980 ng/mL) for peimine and peiminine. All the stock working solutions and QC samples were maintained at −20 °C refrigerator before they were analyzed.

### 3.5. Animal Experiments and Preparation of Plasma Samples

Blood samples were from 12 male beagle dogs (body weight 13–15 kg, divided into two groups, six dogs in each group) were obtained at different time points after oral administration of *Fritillaria ussuriensis* Maxim (1.0 g/kg) and *Fritillaria thunbergii* Miq (1.0 g/kg). The dogs were fasted for 12 h before administration, and had free access to water during the experiment. The blood samples (1.5 mL) of each dog were collected into 5 mL heparinized tube at several specific time points (0, 0.25, 0.5, 1.0, 1.5, 2.0, 2.5, 3.0, 4.0, 6.0, 8.0, 12.0, and 24.0 h) after oral administration. The blood samples were centrifuged at 3500 rpm for 10 min at 4 °C, then the plasma transferred into 2 mL Eppendorf tubes immediately and stored at −20 °C in a refrigerator until analysis.

The sample preparation steps were as follows: 100 μL plasma samples, 10 μL of the IS (1530 ng/mL), and 200 μL methanol were mixed in a 10-mL glass tube and the mixed solutions were ultrasonically shocked for 3 min and then vortex-mixed for 1 min. The mixed samples were subjected to liquid–liquid extraction with 2 mL ethyl acetate, ultrasonically shocked for 3 min and mixed vortex for 3 min, then the samples were centrifuged at 3500 rpm for 5 min to remove particulates. The upper organic layer was removed and evaporated to dryness under a stream of nitrogen gas at 40 °C. Then 100 μL of methanol were used to redissolve the residue and the sample was vortexed for 1 min. The treated samples were transferred into 1.5 mL Eppendorf tubes then centrifuged at 13,000 rpm for 5 min at 4 °C and filtered through a 0.22 μm membrane. A 2-μL sample solution aliquot was injected into the UPLC-MS/MS system.

### 3.6. Validation of the UPLC-MS/MS Method

The developed UPLC-MS/MS method validation was based on the Food and Drug Administration (FDA) guidelines. Accordingly, different validation parameters were evaluated, in terms of specificity and selectivity, linearity, intra-day and inter-day precisions, accuracy, matrix effect, extraction recovery, and stability [32].

#### 3.6.1. Selectivity

Selectivity is the ability of an analytical method to observe whether there has been interference from endogenous substances and other components in plasma at the retention time of the two analytes [33]. The selectivity of the method was evaluated by comparing the chromatograms of blank plasma from 12 beagle dogs with the plasma samples after oral administration. Comparing and analyzing blank samples, plasma samples with IS and the two analytes at LLOQ level, the blank plasmas with I.S. and analytes at low QC levels (1.976 ng/mL for peimine, 1.960 ng/mL for peiminine) and the plasma samples collected 2.0 h after oral administration of **FU** and **FT** powder, there were no detectable interfering peaks at the respective retention time of quantification of the two analytes (LLOQ). 

#### 3.6.2. Linearity and Lower Limits of Quantification

The linearity of each calibration curve was evaluated by plotting the peak area ratios (*y*) of the analyte to the I.S. versus the nominal concentration (*x*) using a linearly weighed (1/*x*^2^) least square linear regression method in duplicate. Calibration curves were considered acceptable when the correlation coefficient (*r*) was greater than 0.99. The LLOQ of peimine and peiminine was defined as the minimum concentration point of the calibration curves, and we measured of the LLOQ precision, expressed as the relative standard deviation (RSD). The calibration curves of two analytes (*S/N* ≥ 10) could be quantified with RSD not exceeding 20%.

#### 3.6.3. Precision and Accuracy

The precision and accuracy of peimine and peiminine were valued by assessing six replicates of the QC samples at four different concentration levels (0.988/1.976/39.52/197.6 ng/mL for peimine and 0.980/1.960/39.20/196.0 ng/mL for peiminine, respectively) on the same day to determine the intra-day precision and on three consecutive validation days to evaluate the inter-day precision. The intra-day and inter-day precision were defined by relative standard deviation (RSD), which was not required to exceed 15% and the accuracy was assessed by relative error (RE) with requirement of within 85–115% except for LLOQ. The accuracy of LLOQ should be within 80–120% and less than 20% of precision.

#### 3.6.4. Extraction Recovery and Matrix Effect

The extraction recovery of peimine and peiminine were determined by analyzing the plasma samples at three different concentrations QC levels (1.976, 39.52 and 197.6 ng/mL for peimine, 1.960, 39.20 and 196.0 ng/mL for peiminine) in six replicates, respectively. The recovery of the IS was determined in sets of five replicates quality control samples at a concentration of 1530 ng/mL. Each compound recovered after extraction and was calculated by comparing the peak areas obtained from extracted samples with post-extracted spiked samples. To evaluate the matrix effect, the same procedure as per evaluation of extraction recovery was repeated but with the exception that recovery calculations were based on using processed standard samples (without plasma) as a reference [34].

#### 3.6.5. Stability

The stability of two analytes in beagle dog plasma was detected under various conditions by analyzing three replicates of plasma samples at three QC levels. Freeze–thaw stability was investigated after three freeze (−20 °C)–thaw (room temperature) cycles. The short-term stability was assessed by analyzing QC plasma samples kept at room temperature for 4 h. The long-term stability was assayed by using QC plasma samples storage at −20 °C for two weeks. The post-preparation stability was determined by analyzing the extracted QC plasma samples kept for 24 h after sample preparation at 4 °C. The concentrations following storage obtained by the calibration curve were compared with the concentrations of freshly prepared standard samples of the same concentrations. When the percentage of RE was within ±15%, the samples were considered stable.

### 3.7. Application to Pharmacokinetic Study

The pharmacokinetics parameters of the analytes were calculated with DAS 2.1 (Mathematical Pharmacology Pharmacology Professional Committee of China, Shanghai, China) by the non-compartmental analysis of plasma concentration versus time data. The maximum plasma concentration (*C*_max_) and the time of maximum plasma concentration (*T*_max_) were observed directly from the measured data [35]. The elimination rate constant (*Ke*) was calculated by linear regression of the terminal points in a semi-log plot of the plasma concentration against time. The elimination half-life (*T*_1/2_) was calculated by the formula *T*_1/2_ = 0.693/*Ke*. The area under the plasma concentration–time curve (AUC_0→t_) to the last measurable plasma concentration (*C_t_*) was estimated by the linear trapezoidal rule from 0 to 24 h. The area under the plasma concentration–time curve to time infinity (AUC_0→∞_) was calculated as AUC_0→∞_ = AUC_0→t_ + *C_t_*/*K*e [36].

## 4. Conclusions

This is the first time the simultaneous determination and the pharmacokinetics of peimine and peiminine in beagle dog plasma has been developed after oral administration of *Fritillaria ussuriensis* Maxim and *Fritillaria thunbergii* Miq powder. The UPLC–MS/MS assay method established in this research provided adequate recovery and matrix effect with great precision and accuracy. Compared with previously reported methods, the present method employed a simple and rapid extraction procedure for sample preparation, and offered higher sensitivity and specificity. In this study, theophylline was used as the internal standard and exhibited a stable response, effective separation, and lower LLOQ of peimine and peiminine than before (1 ng/mL) [31]. Overall, the metabolic trends of *Fritillaria ussuriensis* Maxim and *Fritillaria thunbergii* Miq in beagle dogs are similar. The pharmacokinetic parameters of the two analytes show a process of slow absorption and elimination. The pharmacokinetic results provide useful references for further research on the action mechanisms and pharmacodynamics study of *Fritillaria ussuriensis* Maxim and *Fritillaria thunbergii* Miq.

## Figures and Tables

**Table 1:** Mass spectrometric parameters of peimine, peiminine, and theophylline in MRM mode.

Analytes	Precursor Ion (*m*/*z*)	Product Ion (*m*/*z*)	Qualifier Ion (*m*/*z*)	Fragment (V)	Collision Energy (V)	Polarity
Peimine	432.4	414.4	398.3	124	54	Positive
Peiminine	430.4	412.3	396.3	101	51	Positive
theophylline	181.2	124.1	96.1	62	27	Positive

**Table 2:** The regression equations, correlation coefficients and linear ranges for the determination of the analytes in beagle dog plasma.

Analytes	Regression Equation	*R*	Linear Range (ng/mL)
Peimine	*Y* = 0.0843 *X* + 0.0127	0.9988	0.988–197.6
Peiminine	*Y* = 0.0871 *X* − 0.0502	0.9990	0.980–196.0

**Table 3:** Precision and accuracy of determination of peimine and peiminine in beagle plasma (*n* = 18, six replicates per day for three days).

Compounds	Spiked Concentration (ng/mL)	Measured (ng/mL)	Intra-Day Precision RSD (%)	Inter-Day Precision RSD (%)	Accuracy RE (%)
Peimine	0.988	0.990 ± 0.28	7.40	13.80	5.20
	1.976	1.977 ± 0.07	9.31	13.28	−1.54
	39.52	39.56 ± 1.64	10.40	3.77	−5.57
	197.6	197.8 ± 7.71	14.35	12.23	4.95
Peiminine	0.980	0.9881 ± 0.14	13.30	14.40	−1.20
	1.960	1.965 ± 0.37	14.47	9.98	−3.33
	39.20	39.97 ± 2.05	9.50	11.19	7.37
	196.0	197.3 ± 10.94	8.94	6.01	6.04

**Table 4:** Extraction recoveries and matrix effects for the analytes and I.S. in beagle dog plasma (*n* = 6).

Analytes	Spiked Concentration (ng/mL)	Extraction Recovery		Matrix Effect	
		Mean (%)	RSD (%)	Mean (%)	RSD (%)
Peimine	1.976	85.09	7.74	98.74	7.61
	39.52	82.56	8.07	93.34	9.86
	197.6	84.87	5.52	95.87	4.49
Peiminine	1.960	88.71	9.56	92.06	5.87
	39.20	83.08	7.87	99.34	7.13
	196.0	85.43	3.35	101.2	4.23
IS	1530	96.57	10.64	97.36	4.76

**Table 5:** Stabilities of peimine and peiminine in beagle plasma under various conditions (*n* = 6).

Analytes	Concent-Ration (ng/mL)	Mean ± SD (ng/mL)	Stability (% RE)			
			Three Freeze-Thaw	Short-Term	Long-Term	Post-Preparation
Peimine	1.976	1.986 ± 0.21	4.50	4.06	7.87	6.25
	39.52	40.77 ± 1.29	−1.65	−1.34	0.91	−2.98
	197.6	335.1 ± 7.71	2.19	−4.02	−5.12	−6.34
Peiminine	1.960	1.969 ± 0.37	−5.36	4.10	−3.97	6.06
	39.20	39.96 ± 2.05	11.22	7.97	10.55	12.20
	196.0	196.2±10.94	−1.06	8.76	−6.03	6.53

RE is expressed as (measured concentration/freshly prepared concentration^−1^) × 100%.

**Table 6:** Pharmacokinetic parameters of two constituents after oral administration of **FU** and **FT** powder (mean ± SD, *n* = 12).

Parameters	Analytes			
	*Fritillaria ussuriensis* Maxim		*Fritillaria thunbergii* Miq	
	Peimine	Peiminine	Peimine	Peiminine
*C*_max_ (ng/mL)	68.80 ± 1.63	43.80 ± 4.08	156.89 ± 0.99	56.32 ± 0.83
*T*_max_ (h)	1.83 ± 0.72	2.08 ± 0.21	1.91 ± 0.42	2.17 ± 0.32
*K_e_*	0.230 ± 0.02	0.156 ± 0.03	0.246 ± 0.05	0.234 ± 0.01
*T*_1/2_ (h)	3.012 ± 0.16	4.453 ± 0.35	2.817 ± 0.11	2.965 ± 0.06
AUC_0-t_ (ng/h/L)	340.1 ± 16.86	323.7 ± 39.19	759.4 ± 11.12	319.8 ± 12.54
AUC_0-∞_ (ng/h/L)	367.2 ± 22.35	370.1 ± 49.34	808.2 ± 14.91	342.1 ± 16.11
